# Application of Box-Behnken design for optimization of phenolics extraction from *Leontodon hispidulus* in relation to its antioxidant, anti-inflammatory and cytotoxic activities

**DOI:** 10.1038/s41598-022-12642-2

**Published:** 2022-05-25

**Authors:** Noha Mokhtar Abd-El-Aziz, Mohamed S. Hifnawy, Ahmed A. El-Ashmawy, Rehab A. Lotfy, Inas Y. Younis

**Affiliations:** 1 Department of Medicinal and Aromatic Plants, Desert Research Center, Cairo, Egypt; 2 Department of Pharmacognosy, Faculty of Pharmacy, Cairo University, El Kaser El-Aini, Cairo 11562 Egypt; 3 Medicinal and Pharmaceutical Chemistry Department, Pharmaceutical and Drug Industries Research Institute, National Research Centre, 33 EL Bohouth St. (Former EL Tahrir St.), Dokki, P.O.12622, Giza, Egypt

**Keywords:** Biochemistry, Cancer, Drug discovery, Plant sciences, Medical research, Chemistry

## Abstract

To the best of our knowledge, there have been no phytochemical studies concerning the wild plant *Leontodon hispidulus* Boiss. (Asteraceae). Optimization of the green extraction process of the plant aerial parts, identification of main phenolic compounds, evaluation of antioxidant, anti-inflammatory and anticancer activities of the optimized extract have been carried out. HPLC-analysis was performed using 95% ethanolic extract. 3-Level Box-Behnken Design was applied for optimization of extraction yield and total phenolic content using 3-factors (ethanol/water ratio, material/solvent ratio and extraction time). Antioxidant, anticancer and anti-inflammatory activities were evaluated by ABTS-assay, prostate and cervical carcinoma human cell lines and carrageenan-induced rat paw edema model, respectively. HPLC-analysis showed the presence of quercetin, rutin, kaempferol, chlorogenic and *ρ*-coumaric acids. Increasing both ethanol/water ratio and material/solvent ratio decreased the yield, while, it increased by prolongation of the extraction time. High material/solvent ratio increased the phenolic content. The optimized extract showed high total phenolic content (104.18 µg/mg) using 201 ml of 74.5% ethanol/water at 72 h and good biological activities. Antioxidant activity was found to be 41.89 mg Trolox-equivalent/gm, with 80% free radicals inhibition. For anti-inflammatory activity, 100 mg/kg of the extract inhibited the edema in rats by 83.5% after 4 h of carrageenan injection as compared to 81.7% inhibition by indomethacin. Prostate carcinoma cell line was more sensitive to the anticancer activity of the extract than cervical carcinoma cell line (IC_50_ = 16.5 and 23 μg/ml, respectively). The developed extraction procedure proved to be efficient in enriching the extract with phenolic compounds with promising anticancer, anti-inflammatory and antioxidant activities.

## Introduction

*Leontodon* genus is a characteristic member of the Asteraceae family, it includes about 50 species that are geographically distributed through the Mediterranean, European, and Asian countries^1^. *Leontodon hispidulus* Boiss. is a member of the genus *Leontodon* that grows as a wild plant in Egypt. It is stemless with rigid taproot, rosette leaves and orange-yellow flowers^2^. Sesquiterpenoids especially Guaiane type compounds are the unique chemotaxonomic markers of *Leontodon* in the flower head of *L. autumnalis* and the root of *L. hispidus*^3,4^. Hypocretenolide glycoside was first isolated from *L. hispidus*, which had a potent cytotoxic activity^5^ and anti-inflammatory activity^6^. In addition, other secondary metabolites have been reported in *Leontodon* taxa like phenolic acids (chicoric, chlorogenic, caffeoyl tartaric and 3,5-dicaffeoyl quinic) as well as luteolin and its glycosides^7,8^ which were reported to be natural antioxidants^9^ and were the focus of our study. To the best of our knowledge, there is a scarce data focused on the optimization of the extraction of phenolic compounds from *L. hispidulus*. Therefore, phytochemical screening and HPLC analysis were performed for the first time to allow quantitative measurements of the active metabolites along with the systematic approach to optimize the extraction process. Also, several biological studies were performed for the first time including the antioxidant activity using ABTS assay and the anticancer activity against prostate and cervical carcinoma human cell lines. Moreover, the anti-inflammatory activity was investigated using carrageenan-induced rat paw edema model.

Wild plants proved to be rich in biologically active secondary metabolites. The extraction of active metabolites from the wild plants is greatly affected by several factors viz. extraction time, solvents and plant material/solvent ratio. Hence, it is important to select a proper method of extraction of the bioactive metabolites.

Design of experiment (DOE) is a selective technique to investigate the significance of the different factors affecting a certain process, the relationship between these factors and their effect on the results of this process. Advantages of DOE include finding the best response in the design matrix and determination of the interaction of the factors. This is achieved through running less number of experiments, hence saving time, money, and effort, making it easier to interpret experiments. Response surface methodology (RSM) is an effective analytical function that employs the DOE to predict the conditions leading to optimal response, therefore the optimization process based on RSM is very fast with no need for more experiments or simulations^10,11^.

The optimization process of the extraction conditions is a complicated experiment that needs to be investigated statistically by an efficient technique such as RSM.

Optimization of the extraction of biologically active compounds in bioprocessing, food engineering and pharmaceuticals applied Box-Behnken Design (BBD)^12–14^.

BBD is a second-order design, rotatable or nearly rotatable using a three-level fractional factorial design^15^. It has many advantages including blocks usage, lack of model fit detection, the ability to build sequential designs and estimation of the parameters of the quadratic model. The most important advantage over other designs is that it avoids performing experiments under extreme conditions, like using the lowest or highest levels of all the factors simultaneously which could be expensive or impossible to test^16^.

Inflammation is a protective immune response triggered by damage to the skin or by toxic compounds and pathogens^17^. Recent reports show that high chronic inflammation can be a predisposing factor for the progression of cancer. It is responsible for 20% of deaths due to cancer. The formation of reactive oxygen species is an essential mechanism through which inflammation induces carcinogenesis^18^. According to "Global Cancer Observatory" (GLOBOCAN), 18.1 million new cases and 9.6 million deaths were reported in 2018 worldwide^19^. In our study, we evaluated the potential activity of the plant against two types of human cancer, prostate and cervical carcinoma. In men, carcinoma of the prostate is the majorly recorded malignancy, leading to many complications and finally mortality. Its early detection could be achieved by screening for serum prostate-specific antigen. The standard primary treatments includes radical prostatectomy, radiation, and androgen deprivation therapy^20^. Unfortunately, patients with an initial response to treatment will relapse within three years with androgen-independent prostate cancer (PC3) which is rapidly fatal^21^. On the other side, cervical carcinoma comes second in the most common types of cancer extremely dangerous to women, and is responsible for the majority of deaths in women, especially who are living in low- and middle-income countries^22^. Human papillomavirus (HPVs) is an ubiquitous small virus that is directly linked to the high incidence of cervical carcinoma^23^.

Generally, cancer treatment includes chemotherapy, radiotherapy, surgery, the transformation of stem cells, photodynamic and immunotherapy. Severe side effects result from such treatments as limited bioavailability, toxicity, nonspecificity, fast clearance and metastasis^24^. There are also many undesirable effects that cancer patients find difficult to tolerate such as nausea, vomiting, anemia, fatigue, hair loss, appetite changes, constipation, bleeding and infection. Considering this, there is a global need to look for more selective active compounds of natural origin. Reports showed that 80% of patients around the world use traditional medicine. Over 60% of natural anticancer agents were clinically approved^24^. Their advantages over the synthetic drugs were mediated through targeting specific mechanisms and multiple sites during cancer growth and progression. For example, they increased the antioxidant activity, inactivated the carcinogens, inhibited the proliferation, induced the apoptosis and enhanced the immunity^25^.

Generally, plant extracts are tested for their cytotoxic activity, followed by bioassay-guided fractionation to isolate and purify the active compounds which are then *in-vivo* tested^25^.

The present study aimed to optimize the yield of the active metabolites, especially phenolics by using RSM employing BBD to investigate the biological activities (anticancer, anti-inflammatory, antioxidant) of the optimized extract of *L. hispidulus* aerial parts. Also establishing green extraction via reduction of the volume of the extraction solvent was one of the main targets.

## Materials

### Plant

Collection of the aerial parts was carried out in March at the flowering stage from the North coast of Egypt. It was kindly identified by Dr. Soad Abdallah Hassan "Professor of flowering plants, Botany Department, Faculty of Science, Ain Shams University, Cairo, Egypt". A voucher specimen of the plant was deposited in the herbarium of "Pharmacognosy Department, Faculty of Pharmacy, Cairo University, Cairo, Egypt" with code number 21-3-17. The plant was air-dried, ground to powder and preserved for further analysis.

### Chemicals and standards

Ethanol (analytical grade) for extraction was purchased from El Gomhouria Company for Trading Pharmaceutical Chemicals and Medical Appliances (GOMAC), Cairo, Egypt. Ethanol (HPLC grade) for HPLC analysis, flavonoids and phenolic acids standards, Folin–Ciocalteau and aluminum chloride reagents, standard TROLOX, ABTS, carrageenan and tween-80 (all analytical grade) were obtained from (Sigma-Aldrich, Germany). Standard doxorubicin was kindly provided by "The National Cancer Institute, Cairo, Egypt", and standard indomethacin was kindly provided by "The National Research Centre, Cairo, Egypt" where the experiments were performed.

### Cell lines for anticancer activity study

Human prostate carcinoma (PC3) and human cervical carcinoma (HELA) cell lines were provided by "The National Cancer Institute, Cairo, Egypt".

### Animals for biological studies

The acute toxicity and acute anti-inflammatory studies were carried out on thirty Albino male mice (22–25 gm) and thirty-six Wistar Albino male rats (170–200 gm), respectively. “The animal house of the National Research Centre, Cairo, Egypt" is the source of the animals. Animals were kept in well-ventilated cages under standardized conditions of air, light and temperature with free access to food and water ad libitum. Animal accommodation to the environment started a week earlier. The study protocol was performed according to the recommendations of the ARRIVE guidelines, after approval from the Ethics Committee of the National Research Centre and following the recommendations for the proper care and use of laboratory animals (NIH publication No. 85–23, revised 1985). Serial Number of the protocol according to the Ethical Committee of Faculty of Pharmacy, Cairo University, Egypt: MP (1841).

## Methods

### Phytochemical screening

Conventional standard phytochemical screening protocols were performed for the detection of different chemical classes in the 95% ethanolic extract of the aerial parts including carbohydrates, flavonoids, anthraquinones, coumarins, alkaloids, tannins, terpenes, saponins, sterols and cardiac glycosides^26^.

### HPLC analysis of the phenolic acids and flavonoids

HPLC analysis was carried out following the method of Kim et al.^27^ with minor modification, using 95% ethanolic extract of the aerial parts. "Agilent Technologies 1100 series liquid chromatograph equipped with an autosampler and a diode-array detector" was established for the chromatographic separation. Eclipse column XDB-C18 (150 × 4.6 µm; 5 µm) connected with C18 guard column (Phenomenex, Torrance, CA) was used along with 0.45 µm acrodisc syringe filter (Gelman Laboratory, MI). The mobile phase consisted of solvent A (acetonitrile) and solvent B (2% v/v acetic acid in water).

Gradient elution was applied following the technique "100% B to 85% B in 30 min, 85% B to 50% B in 20 min, 50% B to 0% B in 5 min and 0% B to 100% B in 5 min" with a total run time of 60 min. The injection volume was 50 µl at a flow rate of 0.8 ml/min. Peaks were detected at 280 nm (for benzoic acid derivatives), 320 nm (for cinnamic acid derivatives) and 360 nm (for flavonoids). Peaks identification was performed by congruent retention times and UV spectra and compared with those of the standards.

Three independent injections were performed to calculate SD.

### Optimization of the plant extraction method

#### Design of experiment

The optimization study was performed employing Box-Behnken Design (BBD, 3-factor, 3-level) based on 3^3^ factorial designs (Design Expert V.8). The design consisted of 17 total runs including 5 center points. Three factors (independent variables) and two responses (dependent variables) were studied. Table 1 compiles the coded and actual levels, while the measured responses and the BBD are demonstrated in Table 3. The factors studied and their levels were selected based on previous studies^28,29^. We performed all the work at room temperature (25° C), to avoid any possible degradation of the active constituents (especially phenolic acids and flavonoids) by heat. The factor (ethanol/water ratio) represents the effect of different polar solvents due to the different ratios (95, 72.5 and 50%).

**Table 1:** Actual and coded levels of the variables studied in BBD.

	Levels used		
	Low (− 1)	Middle (0)	High (1)
**Independent variables**			
X1: ethanol/water ratio%	50	72.5	95
X2: extraction time	24 h	48 h	72 h
X3: material/solvent ratio	1:20	1:15	1:10
**Dependent variables**			
Y1: Yield (gm)			
Y2: Total phenolic content (μg/mg)			

#### Preparation of total extract

The powder of the aerial parts, about (20 g for each run) was extracted by maceration at room temperature^30^ using different ratios of ethanol/water, material/solvent and different extraction times according to the optimization study design demonstrated in Table 3. Solvent evaporation was carried out by Rotatory Evaporator "Buchi®R-300, USA" at 45 °C. The yield (Y_1_) for each run was weighed and the total phenolic content (Y_2_) was evaluated.

### Evaluation of the total phenolic content

Spectrophotometer "Shimadzu UV-1650PC, China" was employed for evaluation of the total phenolics using Folin–Ciocalteau method^31^. The total phenolic content for each run (Y_2_) was quantified, per mg of sample, as micrograms of gallic acid equivalent.

### Evaluation of the total flavonoid content

The total flavonoid content of the optimized extract was measured colorimetrically using aluminum chloride method and "Shimadzu spectrophotometer, UV-1650PC, China"^32^. The flavonoids were quantified, per mg of sample, as micrograms of quercetin equivalent.

### Evaluation of the antioxidant activity

Antioxidant activity was evaluated using Hwang et al. method^14^. Stock solutions of the optimized extract were prepared in different concentrations (100–1000 μg/ml). A stock solution (50 μl) and ABTS^+^ solution (4.95 ml) were allowed to react for one hour in darkness, then analyzed colorimetrically at 734 nm using "Spectro UV–VIS Double Beam, Model UVD-3500, Labomed, Inc. La Cienega Blvd. Los Angeles, CA 90,034 USA". TROLOX was used to build the standard calibration curve. The results were in TROLOX equivalent (TE)/gm.

Calculation of the percent inhibition followed the equation:-

$$ {\text{Inhibition }}\left( \% \right) \, = { 1}00 \, \times \, \left( {{\text{A}}_{{{\text{control}}}} - {\text{ A}}_{{{\text{sample}}}} } \right)/{\text{A}}_{{{\text{control}}}} $$

A_control_ = Control reaction absorbance; A_sample_ = Test extract absorbance.

### Acute toxicity study

The study protocol was performed according to the recommendations of the ARRIVE guidelines, after approval from the Ethics Committee of the National Research Centre and following the recommendations for the proper care and use of laboratory animals (NIH publication No. 85-23, revised 1985). Serial Number of the protocol according to the Ethical Committee of Faculty of Pharmacy, Cairo University, Egypt: MP (1841).

Five test groups (six mice per group) were assigned randomly for the Median lethal dose (LD_50_) study. Five doses of the optimized extract in distilled water (1, 2, 3, 4, 5 gm/kg) were administered to the test groups. After 24 h, mortality rate and toxic symptoms were reported in each group^33^.

### Anti-inflammatory activity study

"Carrageenan-induced rat paw edema model"^34^ was employed for this study. Six groups of rats (six rats per group) were divided according to the type of orally administered dose, as follows:-.

Group one: the blank vehicle (1% tween80/H_2_O); negative control.

Group two: 10 mg/kg indomethacin; positive control.

Groups three to six: different doses of the optimized ethanolic extract (25, 50, 100, 200 mg/kg).

All animals were injected subcutaneously in the left hind paw with carrageenan in distilled water (1% w/v, 0.1 ml/paw) after one hour of oral administration to induce edema. "Plethysmometer, UGO Basile 21,025 Comerio, Italy" was used to measure the paw volume before injection of carrageenan and at 1, 2, 3 and 4 h after injection. Calculation of the edema percent and the inhibition percent followed the equations:

$$ {\text{Edema }}\% \, = \frac{{{\text{V}}_{{\text{t}}} {-}{\text{ V}}_{{\text{o}}} }}{{{\text{V}}_{{\text{o}}} }} \times { 1}00 $$

(V_ο_) = paw volume before carrageenan injection (ml); (V_t_) = paw volume at (t) hour after carrageenan injection (ml)

$$ {\text{Inhibition }}\% \, = \frac{{{\text{E}}_{{\text{c}}} {-}{\text{ E}}_{{\text{t}}} }}{{{\text{E}}_{{\text{c}}} }} \times {1}00 $$

(E_c_) = edema rate of control group; (E_t_) = edema rate of treated group.

### Anticancer activity study

Evaluation of the anticancer activity followed the Sulfo-Rhodamine B (SRB) assay^35^. The survival curve "the relation between the optimized extract in concentrations (0, 5, 12.5, 25 and 50 μg/ml) and the surviving fractions" was plotted for PC3 and HELA cell lines. ELISA reader "Sunrise, TECAN, Inc, USA" was employed to measure the color intensity.

### Ethical approval

We confirm that the manuscript has not been submitted to any other journal for publication. All authors have read and approved this version of the article. Approval for the experimental studies has been granted from the Research Ethics Committee for Experimental and Clinical Studies at Faculty of Pharmacy, Cairo University, Cairo, Egypt. The serial number of the protocol: MP (1841). And following the Arrive Guidelines. I also undertake that the article represents valid work. Neither this article nor any part of it has been copied or plagiarized from other works. That the article hasn't been published so far OR communicated OR in simultaneous consideration to some other journals. The Editor of the Journal is empowered to make such editorial changes as may be necessary to make the Article suitable for publication.

## Results and discussion

### Preliminary phytochemical screening

Phytochemical screening results revealed the presence of certain classes of secondary metabolites like carbohydrates, sterols, flavonoids, coumarins, tannins and terpenes in *L. hispidulus* aerial parts. The results also revealed the absence of alkaloids, anthraquinones, saponins and cardiac glycosides.

### HPLC analysis of flavonoids and phenolic acids

**Figure 1:**
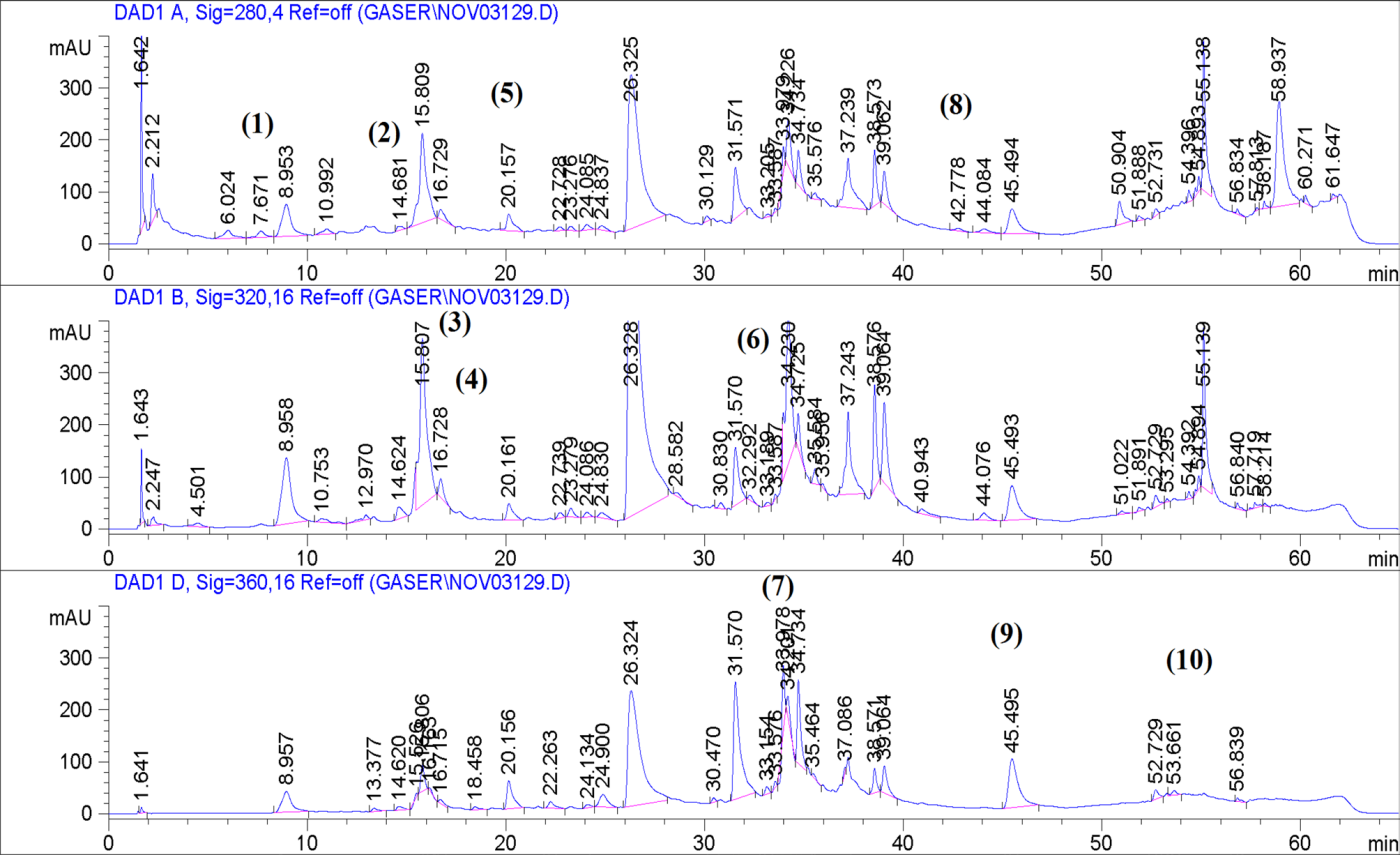
HPLC chromatogram of *L. hispidulus* ethanolic extract.

Table 2 shows the flavonoid and phenolic acid content of *L. hispidulus* aerial parts ethanolic extract. Chlorogenic acid and *ρ*-coumaric acid were the major phenolic acids in *L. hispidulus* extract. Rutin, quercetin, and kaempferol were the main identified flavonoids in the plant extract. HPLC chromatogram is demonstrated in Fig. 1.

**Table 2:** Flavonoid and phenolic acid content of *L. hispidulus* ethanolic extract.

Compound	Retention time (min)	Concentration + SD (µg/g)
1- Protocatechuic acid	7.67	64.155 ± 0.023
2- Catechin	14.68	90.669 ± 0.124
3- Chlorogenic acid	15.8	167.428 ± 0.132
4- Caffeic acid	16.7	39.366 ± 0.092
5- Vanillic acid	20.1	62.521 ± 0.172
6- *p*-coumaric acid	32.2	155.245 ± 0.207
7- Rutin	33.9	173.100 ± 0.229
8- Cinnamic acid	42.7	12.116 ± 0.028
9- Quercetin	45.49	370.049 ± 0.320
10- Kaempferol	53.6	15.197 ± 0.072

### Experimental design

Table 3 demonstrates the BBD matrix where each run of the experiment was represented in a row, with its responses [yield (Y_1_) and total phenolic content (Y_2_)]. Model summary, lack of fit tests and the sequential model sum of squares were calculated for each response and included four models (quadratic, cubic, 2-factor interaction (2FI) and linear). These tests were performed for the determination of the model adequacy to give the maximum yield and phenolic content. The reliability of the model fit is evaluated through the coefficient of variance (CV%), coefficient of determination (R^2^) and adjusted coefficient of determination (R^2^_a_). The effects of the factors and their interaction on each response were evaluated using analysis of variance (ANOVA). For each response, the sequential model sum of squares and lack of fit tests were performed for quadratic, cubic, 2-factor interaction and linear models.

**Table 3:** The Box-Behnken response surface design and corresponding response values.

Std. order	Run	X1 (ethanol/water %)	X2 (extraction time “hr”)	X3 (material/solvent ratio)	Y1 (yield “gm”)	Y2 ± SD (total phenolic “μg/mg”)
8	1	95.00	48	1:10	2.08	87.72 ± 0.14
10	2	72.50	72	1:20	3.65	93.16 ± 0.14
9	3	72.50	24	1:20	3.20	86.66 ± 0.10
11	4	72.50	24	1:10	2.76	102.27 ± 0.10
2	5	95.00	24	1:15	2.70	64.87 ± 0.14
15	6	72.50	48	1:15	3.22	80.89 ± 0.14
7	7	50.00	48	1:10	3.20	77.63 ± 0.24
6	8	95.00	48	1:20	2.63	56.33 ± 0.14
13	9	72.50	48	1:15	3.32	81.54 ± 0.24
12	10	72.50	72	1:10	2.80	103.65 ± 0.14
17	11	72.50	48	1:15	3.26	87.80 ± 0.14
16	12	72.50	48	1:15	3.29	96.98 ± 0.24
1	13	50.00	24	1:15	3.69	72.76 ± 0.24
3	14	50.00	72	1:15	4.16	77.39 ± 0.24
5	15	50.00	48	1:20	3.90	76.17 ± 0.24
4	16	95.00	72	1:15	2.95	65.28 ± 0.14
14	17	72.50	48	1:15	3.30	80.72 ± 0.14

#### The yield (***Y***_1_)

As shown in Table 3, the amount of the yield was ranging from 2.08 gm to 4,157 gm. Table 4 compiles the sequential model fitting data for the yield. The statistical analysis of the sum of squares revealed that the best fitting model was the quadratic, as it was the only model that combined both a significant *p*-value (*p* ˂ 0.0001) as shown in the analysis of the sum of squares and a non-significant lack of fit (*p* = 0.1967, Table 4).

**Table 4:** Sequential model fitting for the yield.

Source	Sum of squares	Mean square	DF	F-value	*P*-value	Remarks
**Sequential sum of squares**						
Mean	172.19	172.19	1			
Linear	3.62	1.21	3	30.98	˂ 0.0001*	
2FI	0.06	0.02	3	0.44	0.7265	
Quadratic	0.43	0.14	3	57.42	˂ 0.0001*	Suggested
Cubic	0.011	3.801E-003	3	2.52	0.1967	Aliased
Residual	6.035E-003	1.509E-003	4			
Total	176.31	10.37	17			
**Lack of fit tests**						
Linear	0.5	0.056	9	36.82	0.0017*	
2FI	0.44	0.073	6	48.66	0.0011*	
Quadratic	0.011	3.801E-003	3	2.52	0.1967	Suggested
Cubic	0.000		0			Aliased
Pure error	6.035E-003	1.509E-003	4			

	Std. Dev	R-Squared	Adjusted R-Squared	Predicted R-Squared	PRESS	
**Model summary statistics source**						
Linear	0.2	0.8773	0.8490	0.7551	1.01	
2FI	0.21	0.8917	0.8267	0.4934	2.09	
Quadratic	0.05	0.9958	0.9903	0.9535	0.19	Suggested
Cubic	0.039	0.9985	0.9941		+	Aliased

*statistically significant: *p* ˂ 0.05.

Analysis of the regression coefficient and *p*-value of each model term were demonstrated in Table 5. The effects of the linear coefficients (A, B and C), two quadratic coefficients (B^2^ and C^2^) and one interactive coefficient (BC) were found to be significant on the yield (*p* ˂ 0.05, Table 5). The highest magnitude of the effect was due to ethanol/water ratio (factor A) with regression coefficient = − 0.57 and a highly significant *p*-value (*p* ˂ 0.0001). The negative sign indicates that it is a decreasing effect (inversely proportional) as the yield decreases with increasing ethanol/water ratio. In other words, increasing the water amount in the ethanol/water ratio has the highest effect on increasing the yield. In addition, the extraction time (factor B) and material/solvent ratio (factor C) both had a highly significant effect on the yield (*p* ˂ 0.0001), but B had a directly proportional effect (regression coefficient = 0.15) while C had an inversely proportional effect (regression coefficient = − 0.32).

**Table 5:** *p*-Values and estimated regression coefficients of the two studied responses.

	Y_1_: yield		Y_2_: phenolic content	
	*p*-value Prob ˃ *F*	Coefficient estimate	*p*-value Prob ˃ *F*	Coefficient estimate
Intercept		3.28		85.59
A-Eethanol/water ratio	˂ 0.0001*	− 0.57	0.1006	− 3.72
B-extraction time	˂ 0.0001*	0.15	0.4387	1.62
C-material/solvent ratio	˂ 0.0001*	− 0.32	0.0072*	7.37
AB	0.0653	− 0.055	0.7157	− 1.05
AC	0.1766	0.037	0.0311*	7.48
BC	0.0045*	− 0.10	0.6594	− 1.28
A^2^	0.3070	− 0.027	0.0002*	− 18.74
B^2^	0.0015*	0.12	0.2722	3.23
C^2^	˂ 0.0001*	− 0.30	0.0262*	7.62

*statistically significant: *p* ˂ 0.05.

Also, two quadratic terms (B^2^ and C^2^) had significant effects (*p*-values 0.0015 and ˂ 0.0001 respectively). While B^2^ had a directly proportional effect (regression coefficient 0.12), C^2^ had an inversely proportional effect (regression coefficient = − 0.3) and was highly significant. Finally, one interactive term (BC) had a significant inversely proportional effect on the amount of the yield (*p*-value = 0.0045 and regression coefficient = − 0.1). Quadratic term (A^2^) and interactive terms (AC and AB) all had non-significant effects based on their *p*-values (*p* > 0.05).

So, simplification of the regression equation after removal of the non-significant terms gave the reduced equation:-

$$ {\text{Yield }} = + {3}.{28 } - 0.{\text{57A }} + 0.{\text{15B }} - 0.{\text{32C }} - 0.{1}0{\text{BC }} + 0.{\text{12B}}^{{2}} - 0.{3}0{\text{C}}^{{2}} $$

**Figure 2:**
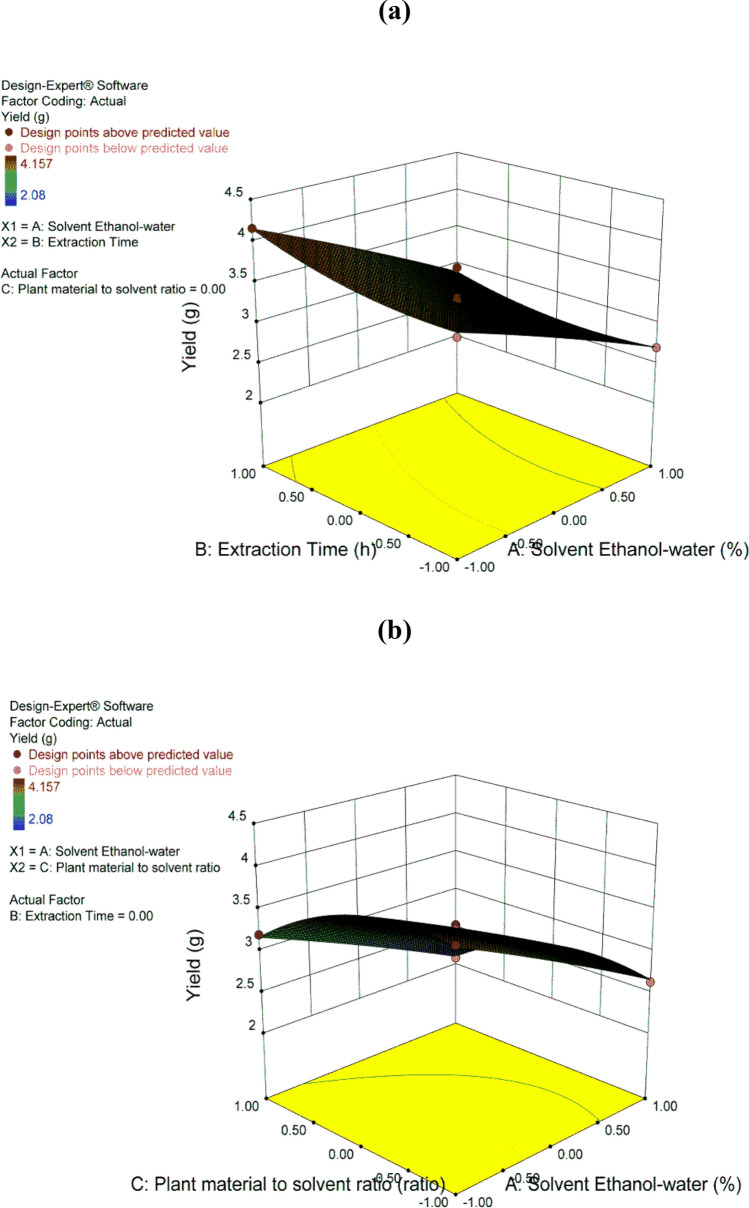
3D response surface plots for the yield (Y_1_). (**a**) Effect of A (ethanol/water ratio), B (extraction time) and their interaction on the yield. (**b**) Effect of A (ethanol/water ratio), C (material/solvent ratio) and their interaction on the yield.

Figure 2 demonstrates the effects of A, B, C and their interactions in the 3D response surface plots which visually represent the significance of the regression equations through illustrating maxima and minima^10^. As shown in Fig. 2a, the yield decreased at the high level of ethanol/water ratio (95%) and at the low level of extraction time (24 h), while it increased at the low level of ethanol/water ratio (50%) and the high level of extraction time (72 h). The highest yield (4.16 gm) was after 72 h of extraction. Figure 2b shows that the yield increased at the low level of ethanol/water ratio (50%) but decreased at the high level (95%). However, there was no significant change in the yield by changing the levels of material to solvent ratio.

#### The total phenolic content (***Y***_2_)

Table 6 compiles the data of the sequential model fitting for the total phenolic content. The range of the total phenolic content was from 56.33 to103.65 μg/mg as demonstrated in Table 3. The quadratic model was chosen as it was the only model that combined both: significant *p*-value of the sum of squares analysis (*p* = 0.0011) and non-significant lack of fit (*p* = 0.9345) which is demonstrated in Table 6. Therefore, navigation of the design space for the total phenolic content was described by this model.

**Table 6:** Sequential model fitting for the total phenolic content.

Source	Sum of squares	Mean square	DF	F-value	*P*-value	Remarks
**Sequential sum of squares**						
Mean	1.140E + 005	1.140E + 005	1			
Linear	565.89	188.63	3	1.14	0.3684	
2F	234.96	78.32	3	0.41	0.7494	
Quadratic	1693.57	564.52	3	18.24	0.0011*	Suggested
Cubic	19.84	6.61	3	0.13	0.9345	Aliased
Residual	196.83	49.21	4			
Total	1.167E + 005	6862.46	17			
**Lack of fit tests**						
Linear	1948.37	216.49	9	4.40	0.0837	
2F	1713.41	285.57	6	5.80	0.0553	
Quadratic	19.84	6.61	3	0.13	0.9345	Suggested
Cubic	0.000		0			Aliased
Pure error	196.83	49.21	4			

	Std. Dev	R-Squared	Adjusted R-Squared	Predicted R-Squared	PRESS	
**Model summary statistics source**						
Linear	12.85	0.2087	0.0261	− 0.5618	4234.10	
2F	13.82	0.2954	− 0.1274	− 2.2271	8748.79	
Quadratic	5.56	0.9201	0.8173	0.7695	624.92	Suggested
Cubic	7.01	0.9274	0.7096		+	Aliased

*statistically significant: *p* ˂ 0.05.

It was found that the effects of one linear coefficient (C), two quadratic coefficients (A^2^and C^2^) and one interactive coefficient (AC) were significant on the total phenolic content (Table 5). Only the material/solvent ratio (factor C) effect was significant (*p*-value = 0.0072) and it was directly proportional to the total phenolic content obtained (regression coefficient = 7.37). In other words, increasing the volume of the extraction solvent in relation to the amount of plant material to be extracted will increase the total phenolic content. Two quadratic terms (ethanol/water ratio “A^2^ “ and material/solvent ratio “C^2^”) had significant effects with *p*-values (0.0002 and 0.0262) respectively. While C^2^ had a directly proportional effect (regression coefficient = 7.62), A^2^ had an inversely proportional effect (regression coefficient = − 18.74) and was highly significant. Finally, one interactive term (AC) had a significant directly proportional effect on the total phenolic content with *p*-value = 0.0311 and regression coefficient = 7.48. All the other terms, including 2 linear factors (B and A), 1 quadratic term (B^2^) and 2 interactive terms (AB and BC) had non-significant effects.

Simplification of the regression equation after removal of the non-significant terms gave the following equation:-

$$ {\text{Total phenolic content }} = + {85}.{59 } + {7}.{\text{37C }} + {7}.{\text{48AC }} - {18}.{\text{74A}}^{{2}} + {7}.{\text{62C}}^{{2}} $$

**Figure 3:**
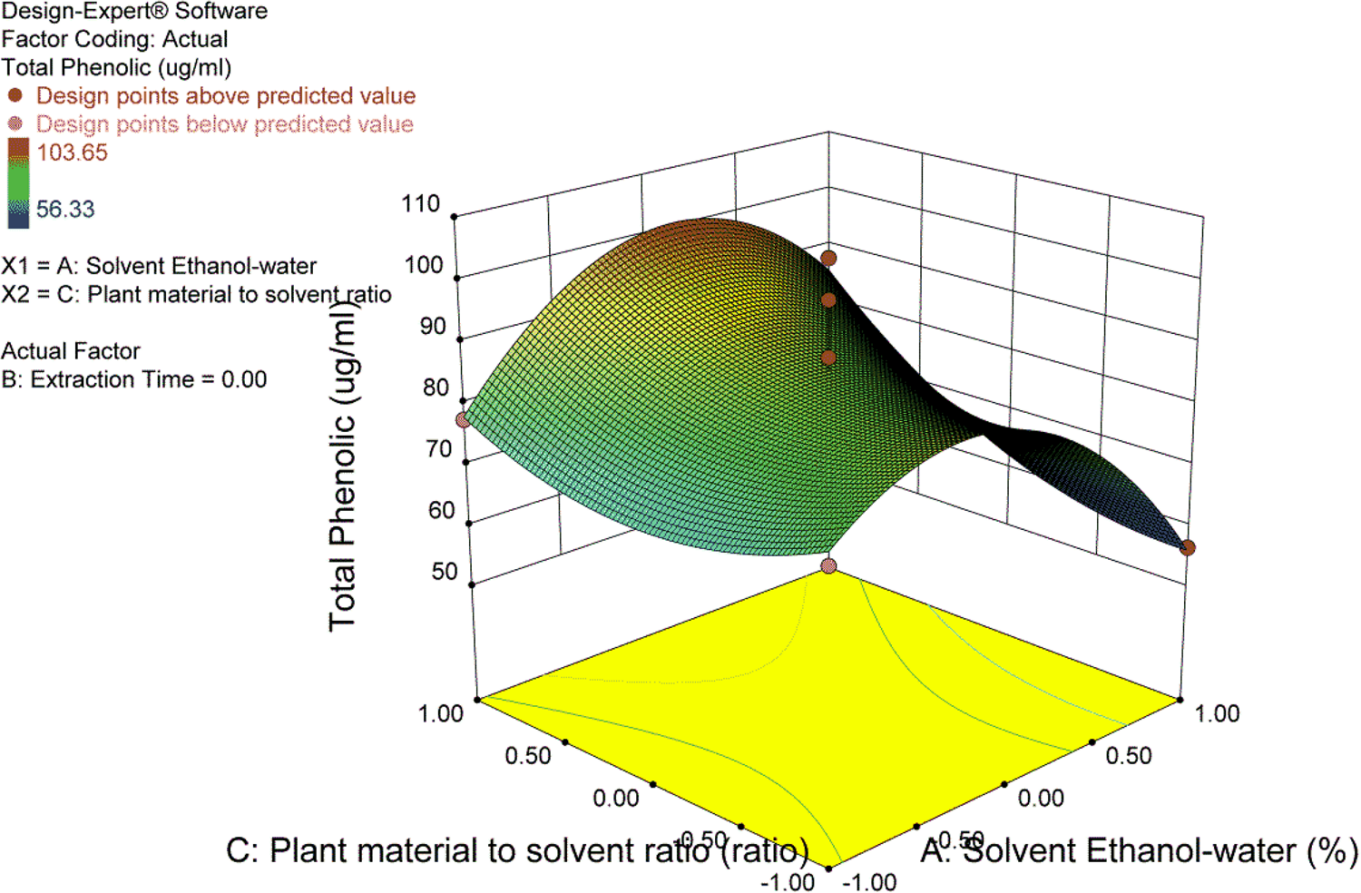
3D response surface plot for the total phenolic content (Y_2_). Effect of A (ethanol/water ratio), C (material/solvent ratio) and their interaction on the total phenolic content.

The effect of C and its interaction with A is visually demonstrated in Fig. 3. The phenolic content decreased at both the high level (95%) and low level (50%) of ethanol/water ratio, while it was slightly changed at the different levels of material/solvent ratio. The middle level of ethanol/water ratio (72.5%) allowed obtaining the highest phenolic content.

**Figure 4:**
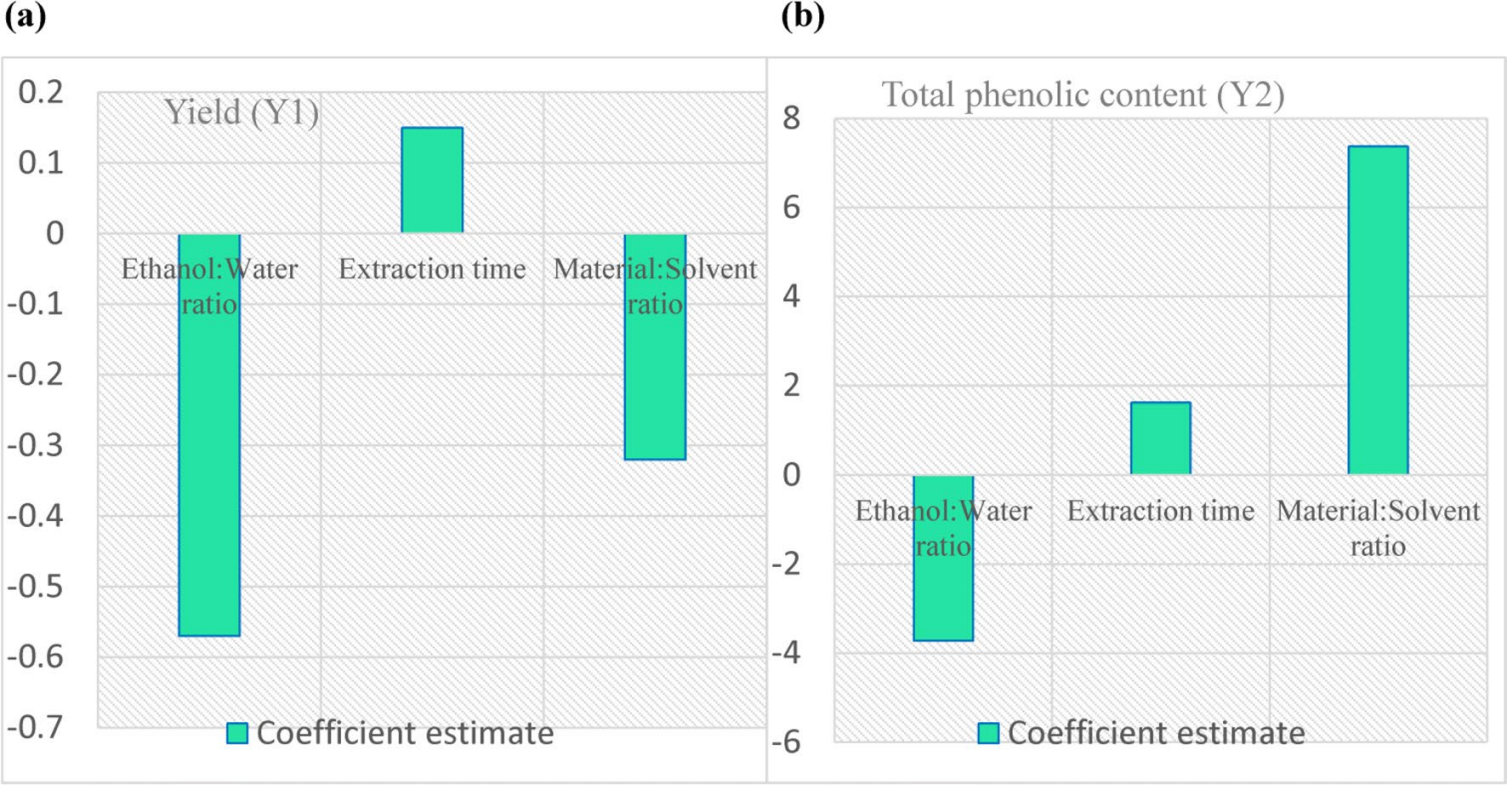
Coefficient estimates of the studied factors for each response. (**a**) Yield (**b**) Phenolic content.

Figure 4 shows the coefficient estimates for the different factors affecting the two responses. Figure 4a shows that both ethanol/water ratio and material/solvent ratio have a decreasing (negative effect) on the amount of the yield which was highly significant. The other significant effect was due to the extraction time but it was an increasing (positive effect). The material/solvent ratio was the only factor that increased the phenolic content significantly (Fig. 4b). While the negative effect of the ethanol/water ratio and the positive effect of the extraction time were both non-significant (*p*-value > 0.05).

#### Model validation and multiple response optimization

**Figure 5:**
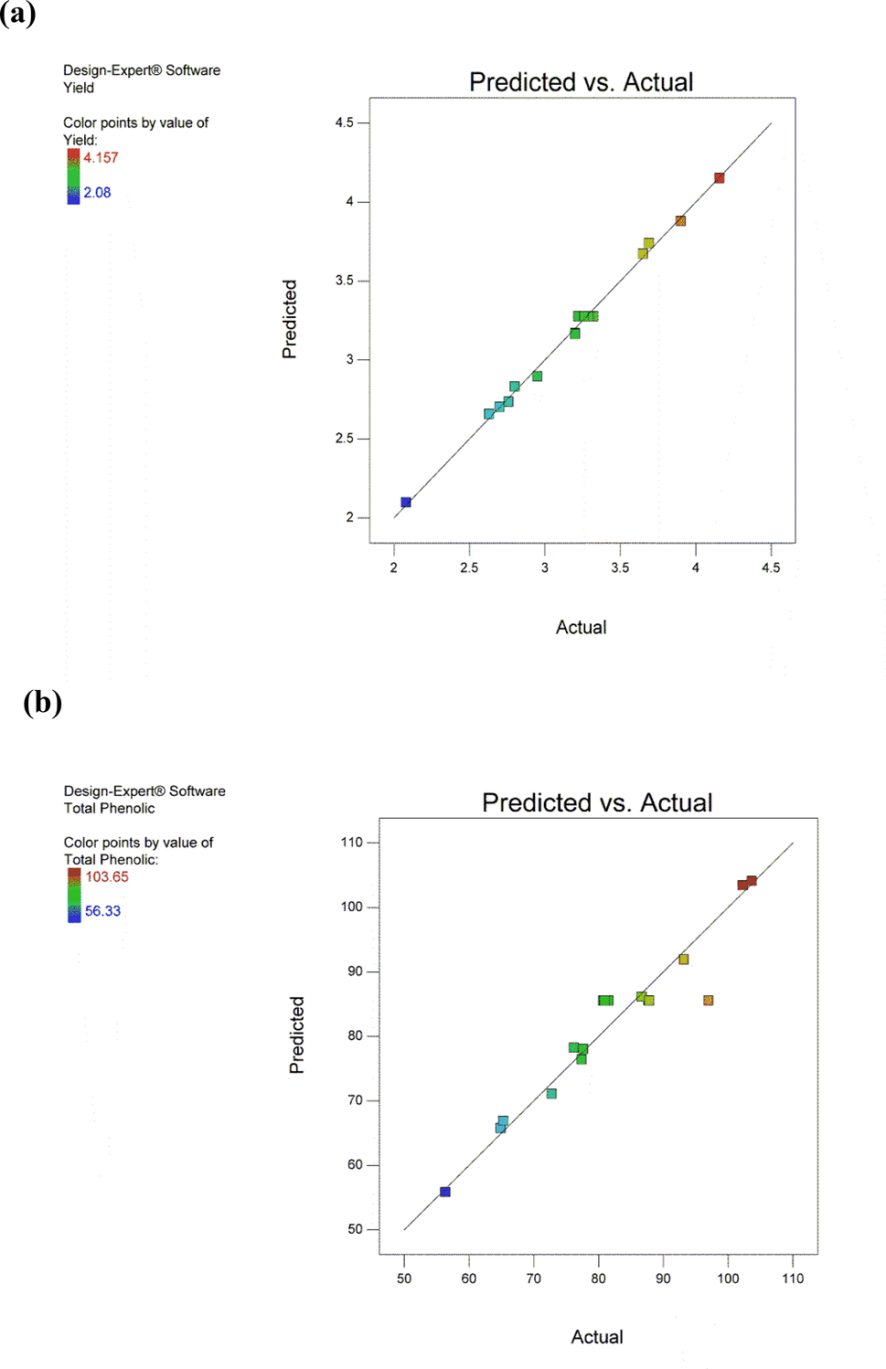
Diagnostic plots for BBD model adequacy. (**a**) Yield (**b**) Phenolic content.

Model accuracy or its capability of prediction (model validation) is demonstrated in Fig. 5, where the predicted values of Y_1_ and Y_2_ were calculated for each run and compared with the experimental values. The figure illustrates that the points on the scatterplots fall very close to the straight line, which means that the actual results obtained were in close agreement with the predicted values for either the yield in Fig. 5a or phenolic content in Fig. 5b. Therefore, the model has a good fit and good prediction capability, and hence, could enhance the relationship between the dependent and the independent variables. Model validation has been also tested via running 3 different extraction runs (checkpoints). The values predicted by the model were in close agreement with the actual results of these checkpoints.

**Figure 6:**
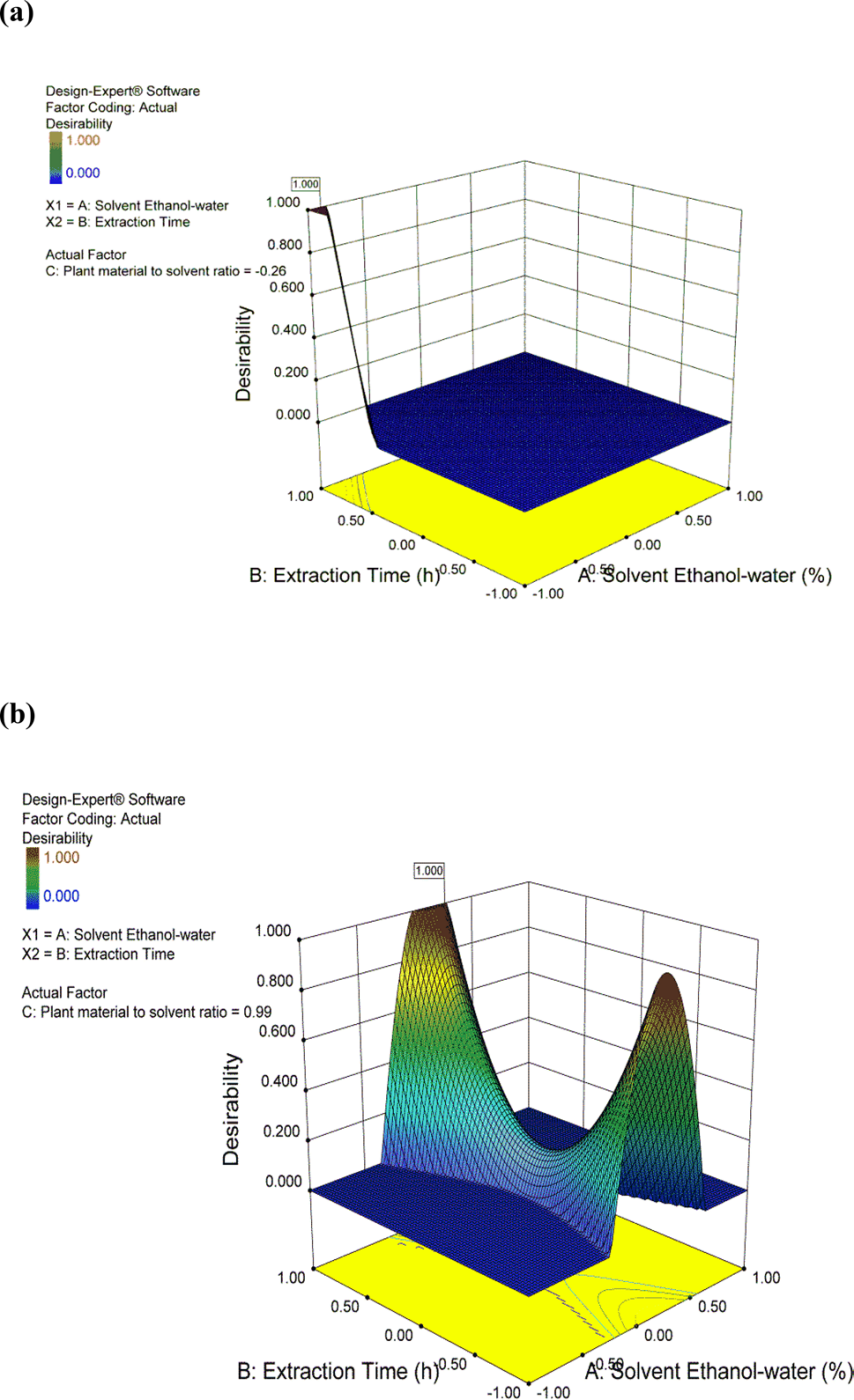
Desirability 3D plots for the optimized extractions. (**a**) (Opt.1) Yield (**b**) (Opt.2) Phenolic content.

A multiple response optimization approach was employed to perform two optimization runs. The first run was performed for maximizing Y_1_ to obtain the maximum yield, while the second was for maximizing Y_2_ to obtain the maximum total phenolic content. In order to optimize 2 responses with different targets, a multi-criteria approach (numerical optimization by desirability function) was employed to estimate the optimum levels for each extraction run^10,36^. The numerical optimization tool in the Deign-Expert is mainly based on the desirability function of the given factors and responses. The desirability function is a transformation of the response variable to a 0–1 scale. The most desirable response will have a desirability of 1, while an undesirable response will have a desirability of 0^37^. The first numerical optimization run (opt.1) was based on maximizing the yield (Y_1_) by putting the three factors (A, B and C) and the other response (Y_2_) in range with the desirability of 1 as shown in Fig. 6a. The second numerical optimization run (opt.2) was based on maximizing the total phenolic content (Y_2_) by putting (A, B and C) and (Y_1_) in range with the desirability of 1 as in Fig. 6b.

The maximum predicted yield (opt.1) was found to be 4.27 gm using 391 ml of 51.125% ethanol at 71.5 h, with desirability equals one, while the maximum predicted phenolic content (opt.2) was found to be 103.9 µg/mg using 201 ml of 74.525% ethanol at 72 h, with desirability equals one, which is demonstrated in Fig. 6. These optimized values were validated using the same conditions to perform the two optimization runs. The actual yield obtained was found to be 4.4 gm (opt.1), while the actual total phenolic content was found to be 104.18 μg/mg (opt.2). The actual results were in close agreement with the predicted values from the optimization analysis using desirability functions, therefore, they confirmed the adequacy of the response model as it achieved the expected optimization. Hence the model is accurate and satisfactory. The percent relative error was calculated as followed^38^:

$$ {\text{Relative error }} = \frac{{{\text{Predicted value }}{-}{\text{ Experimental value}}}}{{\text{Predicted value}}} $$

Table 7 summarizes an excellent correlation between the predicted and actual results of the optimized extraction runs. Validity and practicability of the model were confirmed as the values of the percent relative error were low^36,38^. Therefore, the Box-Behnken design could be successfully used for the optimization of the extraction yield and phenolic content of *L. hispidulus* aerial parts.

**Table 7:** Predicted and experimental values of the optimized extraction responses and their percent relative error.

Response	Predicted values	Experimental values	Percent relative Error (%)
**Opt:1**			
Y_1_	4.27	4.4	− 3.00
Y_2_	85.18	86.12 ± 0.37	− 1.30
**Opt:2**			
Y_1_	2.79	2.83	− 1.40
Y_2_	103.9	104.18 ± 0.37	− 0.27

### Estimation of total phenolics and total flavonoids

The range of the total phenolic content was (56.33 ± 0.14–103.65 ± 0.14 µg/mg). It was estimated using the standard calibration curve of Gallic acid (Y = 0.0041X − 0.0023, R^2^ = 0.9995) for each run as demonstrated in Table 3. While the total flavonoid content for the optimized extract was estimated using the standard calibration curve of quercetin (Y = 0.0063X, R^2^ = 0.999). The highest phenolics concentration was observed in run no.10 (103.65 ± 0.14 µg/mg) in Table 3. The optimized extract produced the maximized concentration of phenolics (104.18 ± 0.37 µg/mg) with minimum percentage of errors (− 0.27%) as shown in Table 7 and flavonoid content (29.73 ± 0.0 μg/mg). Both phenolics and flavonoids are known to be potent natural antioxidant agents^9^.

### Evaluation of the antioxidant activity

This study was performed using ABTS^+^ as a sensitive method for measuring the antioxidant potential of plant metabolites with rapid reaction kinetics^9^. The total optimized extract showed good antioxidant activity of 41.89 mg TE/gm and 80% free radicals inhibition. This was in good agreement with a previous study that reported the promising antioxidant potential of *L. hispidus*^39^. HPLC analysis results confirmed the presence of phenolics, which can be the cause of the antioxidant activity. Quercetin, rutin and chlorogenic acid were the most abundant phenolic compound detected by HPLC analysis. Quercetin and its glycosides were reported to have good antioxidant activity^40^. Moreover, the antioxidant potential of rutin has been well documented, it was reported that rutin caused a significant increase in the levels of the enzymes catalase, glutathione (GSH) and superoxide dismutase (SOD)^41^. Also, chlorogenic acid was reported to inhibit the production of nitric oxide^42^.

### Evaluation of the median lethal dose (LD_50_)

The optimized extract was safe up to 5 g/kg on mice after 24 h since there were no signs of mortality. The test was repeated using the same dose and no mortality or toxicity signs were detected. Therefore, the optimized extract is considered safe according to Globally Harmonized System (GHS) with LD_50_ cut off ˃ 5 g/kg.

### Evaluation of the anti-inflammatory activity

**Figure 7:**
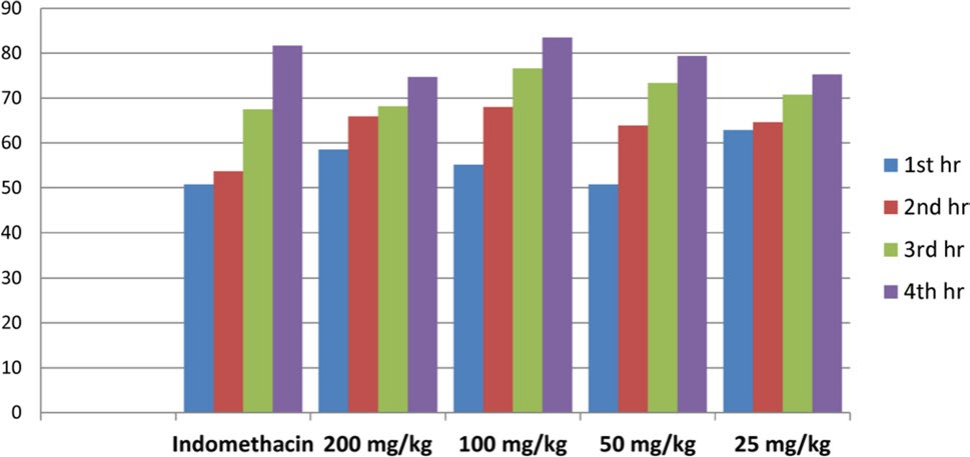
Effect of optimized extract and indomethacin on Carrageenan-induced rat paw edema inhibition.

The anti-inflammatory effects of the optimized extract at different concentrations (200, 100, 50 and 25 mg/kg), and indomethacin (10 mg/kg) are demonstrated in Table 8 and Fig. 7. Interestingly, pretreatment with both the lowest and highest doses (25 and 200 mg/kg) of the optimized extract produced a significant gradual increase of edema inhibition higher than the reference drug indomethacin (10 mg/kg) until 3 h after carrageenan injection. However, after the fourth hour, indomethacin produced higher inhibition.

**Table 8:** Effect of optimized extract and indomethacin (10 mg/kg) on carrageenan-induced rat hind paw edema.

	1st hr		2nd hr		3rd hr		4th hr	
Groups	Edema%	Inhibition%	Edema%	Inhibition%	Edema%	Inhibition%	Edema%	Inhibition%
Carrageenan	116 ± 10.7	0	147 ± 11.2	0	154 ± 9.0	0	170 ± 7.2	0
Indomethacin	57 ± 2.4*	50.8	68 ± 1.9*	53.7	50 ± 1.6*	67.5	31 ± 1.6*	81.7
200 mg/kg	48 ± 4.6*	58.6	50 ± 1.9*	65.9	49 ± 3.6*	68.18	43 ± 4.2*	74.7
100 mg/kg	52 ± 2.8*	55.17	47 ± 2.9*	68.02	36 ± 2.6*	76.6	28 ± 3.3*	83.5
50 mg/kg	57 ± 5.2*	50.8	53 ± 4.6*	63.9	41 ± 3.7*	73.37	35 ± 2.6*	79.4
25 mg/kg	43 ± 3.9*	62.9	52 ± 5.5*	64.6	45 ± 4.4*	70.77	42 ± 2.2*	75.29

*Statistically significant *p* < 0.05.

The dose of 100 mg/kg sustained the inhibitory effect of edema until 4 h after carrageenan injection which was higher than the reference drug indomethacin (10 mg/kg) with a significant difference (*P* ≤ 0.05) in comparison with the control group. These results were in good agreement with a previous study on *leontodon* genus. Hypocretenolide glycoside isolated from *L. hispidus* induced 54% edema inhibition in croton oil-induced mouse ear edema as compared to 40% indomethacin inhibition^6^. There is a direct relationship between oxidative stress and the release of inflammatory mediators. Polyphenolic compounds play a vital role in attenuation of the inflammatory response through their antioxidant activity^17^. In addition, polyphenolics had been reported to exert a potential anti-inflammatory activity through pro-inflammatory mediators suppression (TNF-α and IL-1β)^43^. HPLC analysis of *L. hispidulus* total extract showed the presence of different phenolic compounds which could be responsible for this activity. Chlorogenic acid is one of the top-ranked natural antioxidants through inhibition of Cox-2 expression and attenuation of the pro-inflammatory cytokines (TNF and IL-α)^42^. *P*-coumaric acid was reported to exhibit a promising anti-inflammatory activity against arthritis in rats via the reduction of the circulating immune complexes and TNF expression^44^. Quercetin and its glycosides showed significant anti-inflammatory and antioxidant activity^40^. Interestingly, oral administration of rutin reduced rat paw edema after two hours from carrageenan injection^45^. Kaempferol was reported to have anti-inflammatory activity through reduction of edema induced by croton oil in rats as well as inhibition of leukocytes migration^46^.

### Evaluation of the anticancer activity

**Figure 8:**
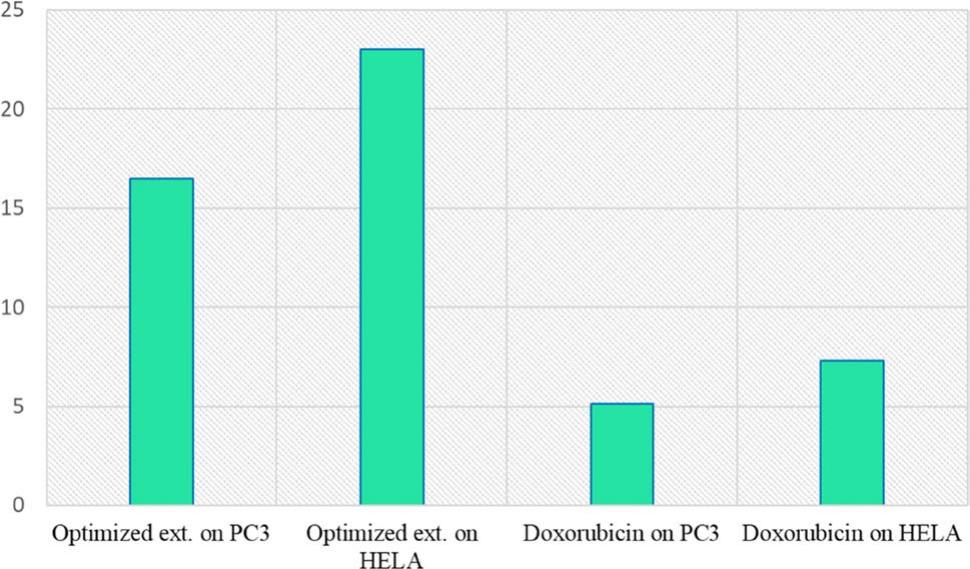
IC_50_ of *L. hispidulus* optimized extract versus doxorubicin.

Hypocretenolide glycoside was isolated from *L. hispidus*, which had a potent anticancer activity more than the control drug Helenalin^5^. To the best of our knowledge, no data was available about the anticancer potential of *L. hispidulus* against both prostate (PC3) and cervical (HELA) carcinoma. Figure 8 demonstrates IC_50_ values of the optimized extract and standard doxorubicin on prostate and cervical carcinoma cell lines.

Prostate carcinoma cell line was more sensitive to the effect of the optimized extract than cervical carcinoma cell line (IC_50_ = 16.5 μg/ml and IC_50_ = 23 μg/ml, respectively). While doxorubicin showed IC_50_ of 5.1 and 7.28 for PC3 and HELA, respectively. Polyphenolic compounds have emerged as promising resources against cancer. As shown from HPLC analysis, the plant contains many phenolic compounds that could be responsible for this activity. Chlorogenic acid as a major component of *Morus nigra* was reported to inhibit prostate carcinoma PC3^47^. Similarly, *ρ*-coumaric acid showed a good cytotoxic activity against gastric cancer^48^ and lung cancer^49^. Moreover, protocatechuic acid and chlorogenic acid demonstrated a promising cytotoxic activity against both colon and liver cancer^50^.

Quercetin was reported to have a direct pro-apoptotic activity on several types of tumor cells, while it showed little or no side effects on normal human cells^51^. The chemotherapeutic activity of rutin was mediated through apoptosis and induction of cell cycle arrest, it also was reported to be a safe anticancer agent with minor side effects^52^. Similarly, the potential activity of kaempferol was mediated through inhibition of metastasis and angiogenesis at relatively low doses without any sign of toxicity^53^. Interestingly, it was also able to decrease the risk of developing skin, liver and colon cancers^54^. The results were in good agreement with a previous study that reported the anticancer activity of *L. hispidulus* against colon carcinoma (HCT-116) and breast carcinoma (MCF-7)^55^.

## Conclusion

Optimization of the plant extraction using Response Surface Methodology and (3^3^) Box- Behnken Design was proved to be a successful method to investigate each factor effect and factors interactions on both the total phenolic content and the yield obtained. The experimental design has been validated and its prediction capability is good. Numerical optimization was performed and the optimized plant extracts containing the maximum yield and the maximum phenolic content showed close agreement between the predicted and experimental values. An optimization study and HPLC analysis for phenolics were performed for the first time on *L. hispidulus*. Furthermore, *in-vivo* anti-inflammatory study and *in-vitro* anticancer study on both prostate and cervical carcinoma cell lines were performed for the first time on *L. hispidulus* total extract. The optimized extract of the total phenolic content showed good *in-vitro* antioxidant activity, promising anticancer activity as well as good anti-inflammatory activity on rats. The plant contains phenolic acids (*ρ*-coumaric and chlorogenic acids) and flavonoids (rutin, quercetin and kaempferol) which could be responsible for the biological activities. Further investigation is necessary to isolate the pure compounds responsible for the biological activities. We believe that this work could be the nucleus for many future studies on *L. hispidulus* and the genus *Leontodon* in general.

## Data Availability

The datasets used and/or analysed during the current study are available from the corresponding author on reasonable request.

## Abbreviations

ABTS^+^: 2,2-Azino-bis (3-ethylbenzothiazoline-6-sulfonic acid)
ANOVA: Analysis of variance
BBD: Box-Behnken design
COX-2: Cyclo-oxygenase enzyme type II
CV%: Coefficient of variance
DOE: Design of experiment
ELISA: Enzyme-linked immunosorbent assay
2FI: Two-factors interaction
GHS: Globally harmonized system
GLOBOCAN: Global cancer observatory
GOMAC: El Gomhouria company for trading pharmaceutical chemicals and medical appliances
GSH: Glutathione enzyme
HCT-116: Human colon carcinoma cell line
HELA: Human cervical carcinoma cell line
HPLC: High-performance liquid chromatography
HPVs: Human papilloma virus
IC_50_: Intermediate toxic dose
IL-α: Interleukin type alpha
IL-1β: Interleukin type one-beta
LD_50_: Median lethal dose
*L. autumnalis*: *Leontodon autumnalis*
*L. hispidus*: *Leontodon hispidus*
*L. hispidulus*: *Leontodon hispidulus*
MCF-7: Human breast carcinoma cell line
NIH: National institutes of health
Opt.1: Optimization run no.1
Opt.2: Optimization run no.2
PC3: Human prostate carcinoma cell line
R^2^: Coefficient of determination
R^2^_a_: Adjusted coefficient of determination
RSM: Response surface methodology
SOD: Superoxide dismutase enzyme
SRB: Sulforhodamine-B assay
TE: Trolox equivalent
TNF-α: Tumor necrosis factor type alpha
TROLOX: 6-hydroxy-2, 5, 7, 8-tetramethylchroman-2-carboxylic acid
UV–VIS: Ultraviole–visible range
